# The prognostic value of the Naples prognostic score for patients with non-small-cell lung cancer

**DOI:** 10.1038/s41598-022-09888-1

**Published:** 2022-04-06

**Authors:** Si-Min Peng, Jin-Jin Ren, Na Yu, Jia-Ying Xu, Guo-Chong Chen, Xiaodong Li, Da-Peng Li, Jing Yang, Zeng-Ning Li, Yu-Song Zhang, Li-Qiang Qin

**Affiliations:** 1 Department of Nutrition and Food Hygiene, School of Public Health, Soochow University, Suzhou, China; 2 Department of Oncology, The Second Affiliated Hospital of Soochow University, Suzhou, China; 3 State Key Laboratory of Radiation Medicine and Protection, School of Radiation Medicine and Protection, Soochow University, Suzhou, China; 4 Department of Oncology, The Third Affiliated Hospital of Soochow University, Changzhou, China; 5 Department of Oncology, The First Affiliated Hospital of Soochow University, Suzhou, China; 6 Department of Clinical Nutrition, The First Affiliated Hospital of Soochow University, Suzhou, China; 7 Department of Nutrition, The First Hospital of Hebei Medical University, Shijiazhuang, China

**Keywords:** Cancer, Biomarkers, Risk factors

## Abstract

The Naples prognostic score (NPS) is an effective inflammatory and nutritional scoring system widely applied as a prognostic factor in various cancers. We aimed to analyze the prognostic value of the NPS in patients diagnosed with non-small-cell lung cancer (NSCLC). We prospectively collected 395 patients diagnosed with NSCLC between January 2016 and December 2018 in two university-affiliated hospitals. Patients were divided into three groups according to their pretreatment NPS (Group 0: NPS = 0; Group 1: NPS = 1–2; Group 2: NPS = 3–4). Kaplan–Meier survival curves indicated that patients with higher NPS had a poorer overall survival (OS) and progress-free survival (PFS) (both *P* < 0.05). NPS was further confirmed as an independent prognostic factors of OS and PFS by multivariable survival analysis (both *P* < 0.05). Furthermore, stratifying by TNM stage, NPS also has significant predictive performance for OS and PFS in both early (I–IIIA) and advanced (IIIB–IV) stage NSCLC (all *P* < 0.05). The time-dependent receiver operating characteristic curve analysis demonstrated that NPS was more superior to other prognostic factors in predicting OS and PFS. In conclusion, NPS may serve as an effective indicator to predict OS and PFS in NSCLC patients regardless of TNM stage.

## Introduction

Lung cancer is the most common cancer type worldwide in terms of both incidence and mortality. Non-small-cell lung cancer (NSCLC) accounts for 80–85% of all lung cancer^1^. Currently, the treatment and prognosis of NSCLC patients are mainly based on TNM staging system^2,3^. However, tumor biology and survival outcomes vary widely among NSCLC patients with the same TNM stage and similar treatment regimens^4,5^, indicating that TNM staging system alone cannot provide sufficient clinical information. Thus, identification of valuable and adequate prognostic indicators is of great significance to further guide individual treatment in NSCLC.

To date, it is widely recognized that the prognosis of cancer patients is closely related to multiple host-related factors in addition to tumor characteristics^6,7^. And an increasing interest focuses on the effect of inflammatory status for the prognosis of patients with malignancies. Cancer-associated inflammation has been implicated in tumor proliferation, promotion of angiogenesis, and metastasis^8^. Systemic inflammatory markers, such as the neutrophil-to-lymphocyte ratio (NLR), lymphocyte-to-monocyte ratio (LMR) and platelet-to-lymphocyte ratio (PLR) have been associated with prognosis in patients with NSCLC^9–11^. Additionally, evidence continues to accumulate associating nutritional status with the short- or long-term prognosis of NSCLC. In particular, some nutritional indicators, such as serum albumin level and total cholesterol, are evaluated to be independently correlated with survival outcome in NSCLC^12,13^. Additionally, several simple scoring system including one or more inflammatory or nutritional parameters, such as prognostic nutritional index (PNI), controlling nutritional status (CONUT) and system inflammation score (SIS) are widely used to predict outcomes^14–16^. However, these predictors remained insufficient due to their limited representation of the general status in cancer patients.

The Naples prognostic score (NPS) was first proposed by Galizia et al. as a novel scoring system for evaluating the prognostic outcomes in colorectal cancer^17^. It consists of four parameters: serum albumin, total cholesterol, NLR and LMR, comprehensively reflecting the patients’ inflammatory and nutritional status. Typically, the NPS is also an independent prognostic factor in endometrial cancer, pancreatic cancer and metastatic colorectal cancer^18–20^. However, the relationship between the NPS and survival has not been completely evaluated in patients with NSCLC. Therefore, here we investigated the prognostic value of the NPS in NSCLC patients.

## Patients and methods

### Patients

The Soochow Lung Cancer Cohort (SLCC) study is an ongoing patient cohort conducted in the first affiliated hospital and the second affiliated hospital of Soochow University. This study was designed to investigate the long-term prognosis factors among lung cancer patients in Southeast China. As described previously^21^, a total of 525 primary lung cancer patients aged ≥ 18 years were enrolled in the cohort between January 2016 and December 2018. For the current study, we excluded patients with small-cell lung cancer (n = 62). We further excluded patients lost to follow-up (n = 29) and those with no sufficient data (n = 39). Finally, the current study included 395 NSCLC patients. This study was approved by the Research Ethics Committee of Soochow University (Approval No. ESCU-2015–0002) and all patients provided written informed consent. We also confirmed that all methods were performed in accordance with the relevant guidelines and regulations.

### Data collection

An in-person interview was conducted by the same trained investigators using a structured questionnaire within 1 week after diagnosis to collect information on demographic factors (e.g., age and gender), disease history [e.g., chronic obstructive pulmonary disease (COPD)], family history of cancer, body mass index (BMI), in addition to behavioral factors such as smoking status and alcohol consumption. Participants were asked to recall the frequency of alcohol consumption during the year prior to the baseline assessment. According to the obtained information, smoking status was categorized as never, former, or current smoker. Current smokers were defined as patients who had smoked continuously or accumulated for at least 6 months and continued to smoke during the survey period or those who gave up smoking for less than 1 year. Former smokers were defined as those who had quit smoking for more than 1 year at the study entry. Furthermore, patients’ medical records were also reviewed to extract their clinical data, including cancer characteristics (TNM stage, histology, lesion and laterality), treatments (surgery, chemotherapy, radiotherapy and targeted therapy), and the levels of various blood analytes. Blood analytes included albumin, total cholesterol, neutrophils, lymphocytes, monocytes, carcinoembryonic antigen (CEA) and neuron-specific enolase (NSE). NLR, LMR, PNI (10 × albumin level + 0.005 × lymphocytes)^14^, CONUT (including albumin, total cholesterol, and lymphocytes)^15^ and SIS (including albumin and LMR)^16^ were calculated based on the collected data.

### Definition of NPS

The definition of NPS was based on the levels of serum albumin, total cholesterol, NLR, and LMR. According to Galizia et al.’s method (the cutoff values of NLR and LMR were defined by MaxStat analysis)^17^, serum albumin level < 40 g/L, total cholesterol level ≤ 180 mg/dL, NLR level > 2.96, or LMR level ≤ 4.44 each was assigned 1 point and otherwise 0 point. The NPS was defined as the sum of the scores of the above parameters. The patients were divided into three groups based on their NPS: 0, 1 or 2, and 3 or 4 (Table 1).

**Table 1:** Calculation of the NPS.

Variable	Cut-off value	Points	NPS group
Albumin (g/L)	≥ 40	0	Group 0: 0 point
	< 40	1	Group 1: 1 or 2 points
Total cholesterol (mg/dL)	> 180	0	Group 2: 3 or 4 points
	≤ 180	1	
NLR	≤ 2.96	0	
	> 2.96	1	
LMR	> 4.44	0	
	≤ 4.44	1	

*LMR* lymphocyte-to-monocyte ratio, *NLR* neutrophil-to-lymphocyte ratio, *NPS* Naples prognostic score.

### Follow-up

Follow up of patient outcomes began at the date of enrollment until the last follow-up date (December 2020) or until the date of death. The survival outcomes were evaluated semiannually. Information sources included the hospital inpatient or outpatient records, patient or family telephonic contact, in addition to local death registration system. Overall survival (OS) was defined as the time from the date of enrollment to the date of death, and progression-free survival (PFS) was defined as the time from the date of enrollment to the date of confirmed progressive disease (PD) or death.

### Statistical analysis

The receiver operating characteristic (ROC) curve was used to determine the optimal cutoff value of the PNI and CONUT. The chi-square test or Fisher’s exact test was applied to explore differences in categorical variables. The Kaplan–Meier method with log-rank test was used to construct survival curves. Univariable and multivariable Cox proportional hazards regression models were used to identify variables associated with OS and PFS. Hazard ratios (HR) and 95% confidence intervals (CI) were then calculated for OS and PFS. Candidate variables with a *P* value < 0.2 on univariable analyses were included in multivariable analysis. Further subgroup analysis was performed according to age, gender, smoking, TNM stage, histology, lesion and surgery, while we also recognized that these analyses are subject to limited statistical power. Interactions were evaluated using the Wald test. Time-dependent receiver operating characteristic curves for the prognostic values of NPS, PNI, CONUT and SIS were estimated using the R package survivalROC. All analyses were performed by IBM SPSS 25.0 and R software version 3.6.3. Two-sided *P* value < 0.05 was considered statistically significant.

## Results

### Association between the NPS and clinical characteristics

Of the 395 patients, there were 252 males and 143 females, with mean age of 63.0 ± 10.5 years (range 24–86 years). According to the NPS system, there were 46 (11.6%) patients at group 0 (NPS 0), 236 (59.8%) at group 1 (NPS 1 or 2) and 113 (28.6%) at group 2 (NPS 3 or 4). And according to the eighth version of UICC/AJCC TNM classification, 138 patients (34.9%) were classified as stage I-IIIA and 257 (65.1%) as stage IIIB-IV. During a median 32 months (range 1–60 months) of follow-up period, 179 (45.3%) patient deaths occurred.

The association between baseline clinical characteristics and the NPS were summarized in Table 2. NPS was significantly associated with age (*P* = 0.038), gender (*P* = 0.020), TNM stage (*P* = 0.011) and surgery (*P* = 0.032). However, no significant differences in smoking, drinking, COPD, family history of cancer, BMI, histology, lesion, laterality, chemotherapy, radiotherapy, targeted therapy, CEA, or NSE were found among the three groups. In addition, patients with higher NPS were more likely to have lower levels of albumin (*P* < 0.001), total cholesterol (*P* < 0.001), LMR (*P* < 0.001) and PNI (*P* < 0.001) while higher levels of NLR (*P* < 0.001), CONUT (*P* < 0.001) and SIS (*P* < 0.001).

**Table 2:** The relationship between NPS and clinical characteristics in NSCLC patients.

Variable	Category	Total (n = 395)	NPS			*P* value
			Group 0 (n = 46)	Group 1 (n = 236)	Group 2 (n = 113)	
Age (years)	< 65	205 (51.9)	32 (69.6)	118 (50.0)	55 (48.7)	0.038
	≥ 65	190 (48.1)	14 (30.4)	118 (50.0)	58 (51.3)	
Gender	Male	252 (63.8)	23 (50.0)	147 (62.3)	82 (72.6)	0.020
	Female	143 (36.2)	23 (50.0)	89 (37.7)	31 (27.4)	
Smoking	Never	200 (50.6)	26 (56.5)	124 (52.5)	50 (44.2)	0.243
	Former/current	195 (49.4)	20 (43.5)	112 (47.5)	63 (55.8)	
Drinking	Yes	84 (21.3)	11 (23.9)	52 (22.0)	21 (18.6)	0.684
	No	311 (78.7)	35 (76.1)	184 (78.0)	92 (81.4)	
COPD	Yes	17 (4.3)	2 (4.3)	12 (5.1)	3 (2.7)	0.578
	No	378 (95.7)	44 (95.7)	224 (94.9)	110 (97.3)	
Family history of cancer	Yes	62 (15.7)	7 (15.2)	37 (15.7)	18 (15.9)	0.994
	No	333 (84.3)	39 (84.8)	199 (84.3)	95 (84.1)	
BMI (kg/m^2^)	< 18.5	34 (8.6)	6 (13.0)	15 (6.4)	13 (11.5)	0.098
	18.5–24	257 (65.1)	24 (52.2)	156 (66.1)	77 (68.1)	
	≥ 24	104 (26.3)	16 (34.8)	65 (27.5)	23 (20.4)	
TNM stage	I-IIIA	138 (34.9)	22 (47.8)	88 (37.3)	28 (24.8)	0.011
	IIIB-IV	257 (65.1)	24 (52.2)	148 (62.7)	85 (75.2)	
Histology	AC	280 (70.9)	35 (76.1)	167 (70.8)	78 (69.1)	0.667
	SCC	99 (25.1)	8 (17.4)	60 (25.4)	31 (27.4)	
	Others	16 (4.1)	3 (6.5)	9 (3.8)	4 (3.5)	
Lesion	Central	137 (34.7)	14 (30.4)	81 (34.3)	42 (37.2)	0.709
	Peripheral	258 (65.3)	32 (69.6)	155 (65.7)	71 (62.8)	
Laterality	Left	171 (43.3)	15 (32.6)	113 (47.9)	43 (38.1)	0.066
	Right	224 (56.7)	31 (67.4)	123 (52.1)	70 (61.9)	
Surgery	Yes	199 (50.4)	28 (60.9)	125 (53.0)	46 (40.7)	0.032
	No	196 (49.6)	18 (39.1)	111 (47.0)	67 (59.3)	
Chemotherapy	Yes	329 (83.3)	38 (82.6)	195 (82.6)	96 (85.0)	0.854
	No	66 (16.7)	8 (17.4)	41 (17.4)	17 (15.0)	
Radiotherapy	Yes	68 (17.2)	5 (10.9)	42 (17.8)	21 (18.6)	0.471
	No	327 (82.8)	41 (89.1)	194 (82.2)	92 (81.4)	
Targeted therapy	Yes	111 (28.1)	12 (26.1)	68 (28.8)	31 (27.4)	0.916
	No	284 (71.9)	34 (73.9)	168 (71.2)	82 (72.6)	
CEA	< 6.5 ng/mL	244 (61.8)	30 (65.2)	150 (63.6)	64 (56.6)	0.404
	≥ 6.5 ng/mL	151 (38.2)	16 (34.8)	86 (36.4)	49 (43.4)	
NSE	< 17 ng/mL	312 (79.0)	38 (82.6)	189 (80.1)	85 (75.2)	0.472
	≥ 17 ng/mL	83 (21.0)	8 (17.4)	47 (19.9)	28 (24.8)	
Albumin	< 40 g/L	136 (34.4)	10 (21.7)	58 (24.6)	68 (60.2)	< 0.001
	≥ 40 g/L	259 (65.6)	36 (78.3)	178 (75.4)	45 (39.8)	
Total cholesterol	≤ 180 mg/dL	205 (51.9)	0 (0)	97 (41.1)	108 (95.6)	< 0.001
	> 180 mg/dL	190 (48.1)	46 (100.0)	139 (58.9)	5 (4.4)	
NLR	≤ 2.96	216 (54.7)	46 (100.0)	167 (70.8)	3 (2.7)	< 0.001
	> 2.96	179 (45.3)	0 (0)	69 (29.2)	110 (97.3)	
LMR	≤ 4.44	299 (75.7)	0 (0)	188 (79.7)	111 (98.2)	< 0.001
	> 4.44	96 (24.3)	46 (100.0)	48 (20.3)	2 (1.8)	
PNI	< 46	116 (29.4)	2 (4.3)	42 (17.8)	72 (63.7)	< 0.001
	≥ 46	279 (70.6)	44 (95.7)	194 (82.2)	41 (36.3)	
CONUT	< 3	288 (72.9)	46 (100.0)	208 (88.1)	34 (30.1)	< 0.001
	≥ 3	107 (27.1)	0 (0)	28 (11.9)	79 (69.9)	
SIS	0	77 (19.5)	36 (78.3)	41 (17.4)	0 (0)	< 0.001
	1	201 (50.9)	10 (21.7)	144 (61.0)	47 (41.6)	
	2	117 (29.6)	0 (0)	51 (21.6)	66 (58.4)	

*AC* adenocarcinoma, *BMI* body mass index, *CEA* carcinoembryonic antigen, *CONUT* controlling nutritional status, *COPD* chronic obstructive pulmonary emphysema, *LMR* lymphocyte-to-monocyte ratio, *NLR* neutrophil-to-lymphocyte ratio, *NPS* Naples prognostic score, *NSE* neuron-specific enolase, *PNI* prognostic nutritional index, *SCC* squamous cell carcinoma, *SIS* system inflammation score.

Values are n (%).

### OS and PFS based on the NPS

**Figure 1:**
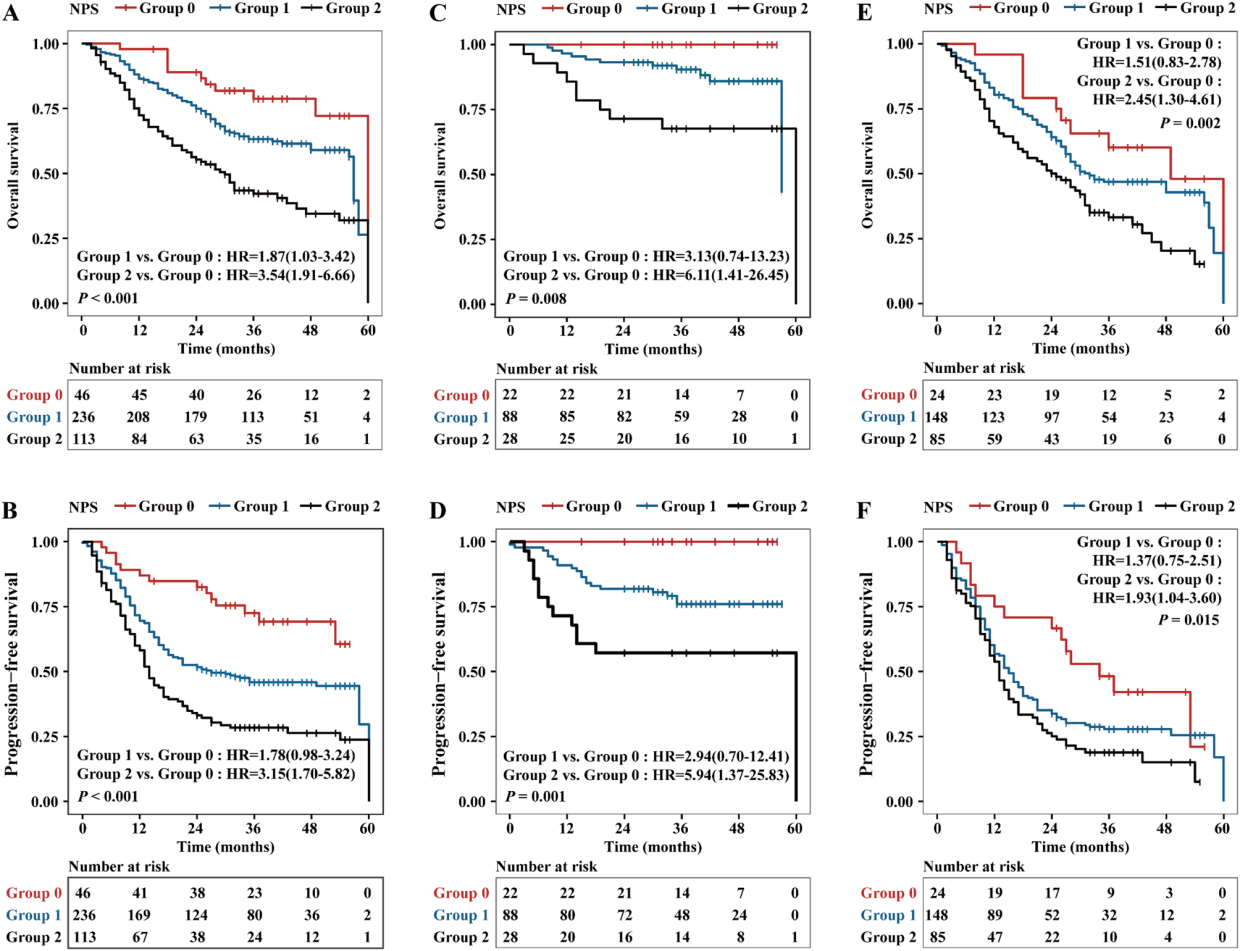
Kaplan–Meier survival curves of Naples prognostic score (NPS) in NSCLC patients. (**A**) Overall survival (OS) in all patients. (**B**) Progression-free survival (PFS) in all patients. (**C**) OS in stage I–IIIA. (**D**) PFS in stage I–IIIA. (**E**) OS in stage IIIB–IV. (**F**) PFS in stage IIIB–IV.

Of the 395 patients, the mean OS time were 37.1, 33.3 and 26.7 months in group 0, 1 and 2, respectively. Three-year OS rates of group 0, 1 and 2 were 73.9%, 58.5% and 38.9% (*P* < 0.001, log-rank test), respectively. Additionally, the mean PFS time were 34.0, 26.1 and 20.2 months in group 0, 1 and 2, respectively. Three-year PFS rates of group 0, 1 and 2 were 69.6%, 44.9% and 26.5% (*P* < 0.001, log-rank test), respectively. The Kaplan–Meier analysis showed that both OS and PFS of patients in group 0 was significantly higher than those of patients in group 1 and group 2 (Fig. 1A,B). We further divided the whole group into stage I–IIIA and stage IIIB–IV subgroups, significant difference was also observed in both OS and PFS based on the NPS regardless of the stage subgroups (Fig. 1C–F).

### Univariable and multivariable analyses of OS and PFS

We assessed the NPS and other possible prognostic factors (age, gender, smoking, drinking, COPD, family history of cancer, BMI, TNM stage, histology, lesion, laterality, surgery, chemotherapy, radiotherapy, targeted therapy, CEA, NSE, albumin, total cholesterol, NLR, LMR, PNI, CONUT and SIS), to predict the OS and PFS of NSCLC patients in present study.

Univariable analysis suggested that NPS, gender, smoking, drinking, COPD, BMI, TNM stage, histology, surgery, CEA, NSE, albumin, total cholesterol, NLR, LMR, PNI, CONUT and SIS were significant prognostic factors for OS. When these factors were included in the multivariable model, the NPS group 2 (HR, 2.57; 95% CI 1.32–4.98; *P* = 0.009) were independent prognostic indicators for unfavorable OS of NSCLC patients. And other independent prognostic factors were smoking (HR, 1.78; 95% CI 1.30–2.45; *P* < 0.001), BMI ≥ 24 kg/m^2^ (HR, 0.39; 95% CI 0.23–0.66; *P* < 0.001), TNM stage (HR, 2.16; 95% CI 1.45–3.21; *P* < 0.001), surgery (HR, 3.08; 95% CI 2.01–4.71; *P* < 0.001) and PNI (HR, 0.68; 95% CI 0.49–0.96; *P* = 0.029) (Table 3).

**Table 3:** Univariable and multivariable analysis of overall survival in NSCLC patients.

Variable	Univariable analysis		Multivariable analysis	
	HR (95% CI)	*P* value	HR (95% CI)	*P* value
Age (vs. < 65)	1.19 (0.88–1.59)	0.254		
Gender (vs. male)	0.62 (0.45–0.86)	0.004	0.64 (0.38–1.06)	0.118
Smoking (vs. never)	1.67 (1.24–2.25)	0.001	1.78 (1.30–2.45)	< 0.001
Drinking (vs. yes)	0.78 (0.55–1.09)	0.141	0.96 (0.64–1.44)	0.639
COPD (vs. yes)	0.62 (0.33–1.18)	0.146	0.61 (0.30–1.25)	0.474
Family history of cancer (vs. yes)	1.21 (0.79–1.87)	0.381		
**BMI (vs. < 18.5 kg/m**^**2**^)				
18.5–24	0.50 (0.33–0.76)	0.001	0.75 (0.48–1.16)	0.194
≥ 24	0.27 (0.16–0.45)	< 0.001	0.39 (0.23–0.66)	0.001
TNM stage (vs. I–IIIA)	4.11 (3.01–5.32)	< 0.001	2.16 (1.45–3.21)	< 0.001
**Histology (vs. AC)**				
SCC	1.48 (1.06–2.05)	0.020	1.39 (0.96–1.99)	0.078
Others	1.40 (0.73–2.68)	0.312	2.05 (1.05–4.02)	0.036
Lesion (vs. peripheral)	1.13 (0.83–1.55)	0.421		
Laterality (vs. left)	0.94 (0.70–1.26)	0.684		
Surgery (vs. yes)	4.84 (3.45–6.79)	< 0.001	3.08 (2.01–4.71)	< 0.001
Chemotherapy (vs. yes)	1.10 (0.74–1.62)	0.652		
Radiotherapy (vs. yes)	0.82 (0.57–1.18)	0.279		
Targeted therapy (vs. yes)	0.82 (0.60–1.12)	0.222		
CEA (vs. < 6.5 ng/mL)	1.69 (1.26–2.26)	< 0.001	1.22 (0.83–1.77)	0.613
NSE (vs. < 17 ng/mL)	1.49 (1.07–2.09)	0.019	1.02 (0.70–1.48)	0.928
Albumin (vs. < 40 g/L)	0.50 (0.37–0.67)	< 0.001	0.85 (0.39–1.88)	0.699
Total cholesterol (vs. < 180 mg/dL)	0.77 (0.57–1.04)	0.087	1.37 (0.83–2.26)	0.157
NLR (vs. < 2.96)	1.79 (1.33–2.40)	< 0.001	0.85 (0.52–1.41)	0.772
LMR (vs. < 4.44)	0.51 (0.35–0.76)	0.001	1.04 (0.60–1.81)	0.434
PNI (vs. < 46)	0.45 (0.33–0.61)	< 0.001	0.68 (0.49–0.96)	0.029
CONUT (vs. < 3)	1.80 (1.32–2.46)	< 0.001	1.34 (0.87–2.04)	0.493
**SIS (vs. 0)**				
1	1.97 (1.24–3.13)	0.004	1.04 (0.54–1.97)	0.849
2	3.39 (2.08–5.51)	< 0.001	1.08 (0.36–3.26)	0.637
**NPS (vs. group 0)**				
Group 1	1.87 (1.03–3.42)	0.040	1.80 (0.97–3.32)	0.062
Group 2	3.54 (1.91–6.55)	< 0.001	2.57 (1.32–4.98)	0.005

*AC* adenocarcinoma, *BMI* body mass index, *CEA* carcinoembryonic antigen, *CI* confidence interval, *CONUT* controlling nutritional status, *COPD* chronic obstructive pulmonary emphysema, *HR* hazard ratio, *LMR* lymphocyte-to-monocyte ratio, *NLR* neutrophil-to-lymphocyte ratio, *NPS* Naples prognostic score, *NSE* neuron-specific enolase, *PNI* prognostic nutritional index, *SCC* squamous cell carcinoma, *SIS* system inflammation score.

With regard to PFS, univariable analysis indicated that gender, smoking, drinking, COPD, BMI, TNM stage, histology, surgery, radiotherapy, targeted therapy, CEA, NSE, albumin, total cholesterol, NLR, LMR, PNI, CONUT and SIS were significant factors for PFS. Subsequent multivariable analysis further demonstrated that per unit increase in NPS (group 1: HR, 2.22; 95% CI 1.19–4.14; *P* = 0.012; group 2: HR, 3.94; 95% CI 1.89–8.21; *P* < 0.001) could independently predict poorer PFS for NSCLC patients. And other independent prognostic factors were smoking (HR, 1.73; 95%CI, 1.27–2.37; *P* = 0.001), BMI (18.5–24 kg/m^2^: HR, 0.60; 95% CI 0.39–0.93; *P* = 0.022; ≥ 24 kg/m^2^: HR, 0.32; 95% CI 0.19–0.55; *P* < 0.001), TNM stage (HR, 1.86; 95% CI 1.22–2.83; *P* = 0.004), histology (others: HR, 3.11; 95% CI 1.58–6.11, *P* = 0.001), surgery (HR, 3.24; 95% CI 2.03–5.17; *P* < 0.001) and total cholesterol (HR, 1.54; 95% CI 1.04–2.30; *P* = 0.033) (Table 4).

**Table 4:** Univariable and multivariable analysis of progression-free survival in NSCLC patients.

Variable	Univariable analysis		Multivariable analysis	
	HR (95% CI)	*P* value	HR (95% CI)	*P* value
Age (vs. < 65)	1.19 (0.89–1.60)	0.243		
Gender (vs male)	0.64 (0.47–0.89)	0.008	0.75 (0.45–1.24)	0.379
Smoking (vs. never)	1.64 (1.22–2.21)	0.001	1.73 (1.27–2.37)	0.001
Drinking (vs. yes)	0.74 (0.53–1.04)	0.081	0.77 (0.51–1.16)	0.135
COPD (vs. yes)	0.62 (0.33–1.17)	0.140	0.64 (0.31–1.31)	0.291
Family history of cancer (vs. yes)	1.26 (0.82–1.94)	0.289		
**BMI (vs. < 18.5 kg/m**^**2**^)				
18.5–24	0.46 (0.30–0.70)	< 0.001	0.60 (0.39–0.93)	0.022
≥ 24	0.25 (0.15–0.42)	< 0.001	0.32 (0.19–0.55)	< 0.001
TNM stage (vs. I–IIIA)	3.83 (2.80–5.23)	< 0.001	1.86 (1.22–2.83)	0.004
**Histology (vs. AC)**				
SCC	1.38 (0.99–1.92)	0.058	1.43 (0.96–2.11)	0.078
Others	1.77 (0.93–3.38)	0.083	3.11 (1.58–6.11)	0.001
Lesion (vs. peripheral)	1.07 (0.78–1.45)	0.685		
Laterality (vs. left)	0.94 (0.70–1.26)	0.673		
Surgery (vs. yes)	4.68 (3.31–6.62)	< 0.001	3.24 (2.03–5.17)	< 0.001
Chemotherapy (vs. yes)	1.03 (0.69–1.52)	0.903		
Radiotherapy (vs. yes)	0.77 (0.53–1.10)	0.152	0.97 (0.64–1.46)	0.958
Targeted therapy (vs. yes)	0.78 (0.57–1.06)	0.110	0.91 (0.62–1.34)	0.964
CEA (vs. < 6.5 ng/mL)	1.73 (1.29–2.32)	< 0.001	1.08 (0.74–1.56)	0.540
NSE (vs. < 17 ng/mL)	1.78 (1.29–2.46)	< 0.001	1.31 (0.91–1.88)	0.138
Albumin (vs. < 40 g/L)	0.55 (0.41–0.74)	< 0.001	1.05 (0.62–1.78)	0.359
Total cholesterol (vs. < 180 mg/dL)	0.81 (0.60–1.09)	0.157	1.54 (1.04–2.30)	0.033
NLR (vs. < 2.96)	1.73 (1.29–2.32)	< 0.001	0.91 (0.55–1.49)	0.686
LMR (vs. < 4.44)	0.57 (0.39–0.83)	0.004	1.01 (0.58–1.76)	0.893
PNI (vs. < 46)	0.47 (0.35–0.64)	< 0.001	1.30 (0.76–2.23)	0.155
CONUT (vs. < 3)	1.68 (1.23–2.29)	0.001	1.18 (0.77–1.80)	0.278
**SIS (vs. 0)**				
1	1.76 (1.11–2.78)	0.016	1.09 (0.58–2.04)	0.511
2	2.79 (1.73–4.49)	< 0.001	1.02 (0.34–3.09)	0.374
**NPS (vs. group 0)**				
Group 1	1.78 (0.98–3.24)	0.060	2.22 (1.19–4.14)	0.012
Group 2	3.15 (1.70–5.82)	< 0.001	3.94 (1.89–8.21)	< 0.001

*AC* adenocarcinoma, *BMI* body mass index, *CEA* carcinoembryonic antigen, *CI* confidence interval, *CONUT* controlling nutritional status, *COPD* chronic obstructive pulmonary emphysema, *HR* hazard ratio, *LMR* lymphocyte-to-monocyte ratio, *NLR* neutrophil-to-lymphocyte ratio, *NPS* Naples prognostic score, *NSE* neuron-specific enolase, *PNI* prognostic nutritional index, *SCC* squamous cell carcinoma, *SIS* system inflammation score.

Furthermore, we stratified NSCLC patients according to age, gender, smoking, TNM stage, histology, lesion and surgery and examined whether the relation between the NPS and OS or PFS was modified by above clinical characteristics. We found that the inverse association of NPS with OS or PFS rate remained in most subgroups with only a few exceptions. In particular, when NSCLC patients were stratified by TNM stage, multivariable analysis indicated that NPS was an independent prognostic factor for OS and PFS in both NSCLC patients staged I–IIIA and staged IIIB–IV. However, smoking significantly modified the relationship between NPS and OS or PFS (*P* for interaction = 0.036 and < 0.001, respectively) (Table 5).

**Table 5:** Subgroup analysis of overall survival and progression-free survival in NSCLC patients.

Variable	Category	Event/total	NPS			*P* for interaction
			Group 0	Group 1	Group 2	
Overall survival*						
Age (years)	< 65	87/205	1	2.01 (0.75–5.40)	4.10 (1.37–12.24)	0.333
	≥ 65	92/190	1	2.71 (1.00–7.35)	3.29 (1.17–9.29)	
Gender	Male	126/252	1	2.85 (1.26–6.42)	3.56 (1.54–8.21)	0.702
	Female	53/143	1	1.68 (0.64–4.40)	3.29 (1.17–9.22)	
Smoking	Never	77/200	1	2.92 (1.15–7.46)	4.28 (1.63–11.20)	0.036
	Former/current	102/195	1	1.37 (0.36–5.22)	5.27 (0.68–40.69)	
TNM stage	I–IIIA	21/138	1	2.01 (0.75–5.42)	10.76 (2.23–51.93)	0.617
	IIIB–IV	158/257	1	2.87 (1.09–7.55)	3.04 (1.10–8.41)	
Histology	AC	118/280	1	2.11 (0.90–4.93)	3.96 (1.66–9.47)	0.344
	SCC	51/99	1	1.86 (0.18–19.57)	0.61 (0.04–10.06)	
Lesion	Central	62/137	1	3.11 (1.24–7.83)	3.84 (1.47–10.00)	0.540
	Peripheral	117/258	1	1.48 (0.29–7.60)	5.08 (0.44–59.22)	
Surgery	Yes	48/199	1	1.45 (0.48–4.38)	4.98 (1.54–16.09)	0.958
	No	131/196	1	2.47 (0.79–7.69)	3.77 (0.79–18.13)	
Progression-free survival*						
Age (years)	< 65	111/205	1	1.77 (0.79–3.97)	3.65 (1.60–8.33)	0.104
	≥ 65	116/190	1	1.32 (0.32–5.44)	1.23 (0.17–8.81)	
Gender	Male	154/252	1	2.28 (1.03–5.04)	3.21 (1.43–7.17)	0.628
	Female	73/143	1	1.93 (0.39–9.65)	5.01 (0.41–61.31)	
Smoking	Never	102/200	1	2.61 (1.03–6.63)	3.21 (1.24–8.33)	< 0.001
	Former/current	125/195	1	1.36 (0.38–4.78)	4.57 (0.67–31.44)	
TNM stage	I–IIIA	33/138	1	1.49 (0.61–3.66)	6.36 (1.44–28.18)	0.835
	IIIB–IV	194/257	1	2.47 (0.99–6.19)	2.88 (1.12–7.45)	
Histology	AC	150/280	1	2.18 (0.94–5.06)	3.32 (1.40–7.86)	0.203
	SCC	67/99	1	1.44 (0.15–13.85)	0.56 (0.04–8.10)	
Lesion	Central	84/137	1	2.89 (1.15–7.26)	3.79 (1.48–9.75)	0.349
	Peripheral	143/258	1	1.37 (0.27–6.89)	3.44 (0.31–37.78)	
Surgery	Yes	72/199	1	1.07 (0.79–2.98)	3.51 (1.19–10.37)	0.945
	No	155/196	1	2.13 (0.97–4.66)	2.82 (1.26–6.27)	

*Adjusted for age, gender, smoking, drinking, COPD, family history of cancer, BMI, TNM stage, histology, lesion, laterality, surgery, chemotherapy, radiotherapy, targeted therapy, CEA, NSE, albumin, total cholesterol, NLR, LMR, PNI, COUNT and SIS and the corresponding variable was removed from the models when it was a stratified factor. *AC* adenocarcinoma, *BMI* body mass index, *CEA* carcinoembryonic antigen, *NSE* neuron-specific enolase, *SCC* squamous cell carcinoma.

### Prognostic value of NPS

**Figure 2:**
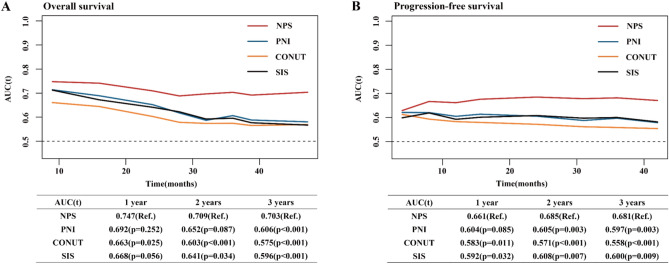
Time-dependent receiver operating characteristic curves of NPS, PNI, CONUT and SIS for prediction of survival. The time dependence of each area under the curve (AUC) for survival is shown at 1, 2 and 3 years. (**A**) Overall survival. (**B**) Progression-free survival. *CONUT* controlling nutritional status, *NPS* Naples prognostic score, *PNI* prognostic nutritional index, *Ref.* reference, *SIS* system inflammation score.

We compared the prognostic value of the NPS with other scoring systems (PNI, CONUT and SIS). The time-dependent ROC curves showed that NPS obtained higher AUCs in dynamic trends compared with other variables within the follow-up time. The AUC of the NPS, PNI, CONUT, and SIS for predicting 3-year OS were 0.703, 0.606, 0.575 and 0.596, while the corresponding AUCs for predicting three-year PFS were 0.681, 0.597, 0.558 and 0.600, indicating that NPS was superior to other scoring systems for predicting long-term survival (Fig. 2). We further compared their prognostic value in different stage subgroups, and NPS remained a higher prognostic performance in both stage I–IIIA and stage IIIB–IV patients (see Supplementary Fig. S1 online).

## Discussion

In the present study, we evaluated the potential importance of NPS as a pre-treatment prognostic indicator for OS and PFS among patients with NSCLC. We identified that NPS had discrimination for long-term survival in NSCLC patients, as high NPS was associated with poor OS and PFS regardless of the patients’ TNM stage. Furthermore, NPS showed more superior prognostic value than previous scoring systems (PNI, CONUT, and SIS) for the prediction of long-term OS and PFS. However, the correlation between NPS and survival in NSCLC may be influenced by smoking status.

Inflammation and nutritional status are closely related to the occurrence and development of cancers^8,22–25^. And an increasing number of studies have explored various inflammatory and nutritional prognostic indicators and scoring systems. Inflammatory indicators such as NLR, LMR and PLR^26–29^, and nutritional indicators such as albumin and total cholesterol have been used to estimate the prognosis of various types of cancers, including lung cancer^12,13^. However, a single marker of inflammation or nutrition cannot fully represent the general status of cancer patients. Furthermore, several studies have also suggested that albumin, total cholesterol, NLR and LMR hold no prognostic value for certain groups of cancer patients^30–33^. In our study, none of the four parameters could be considered as an independent prognostic factor for OS, and only total cholesterol had significant prognostic value for PFS in NSCLC, supporting that the single marker with a limited predictive ability cannot be applied to a wide variety of malignancies. Thus, in recent years, various scoring system integrating one or more above parameters, such as PNI, CONUT and SIS^14–16^, are used to the prognostic assessment of patients with NSCLC. However, their performances of prognosis are still uncertain.

NPS, which is a novel scoring system based on serum albumin, total cholesterol, NLR and LMR, may more comprehensively reflect the host inflammatory and nutritional status. Recent studies have suggested that an elevated level of NPS is related with poor prognosis in patients with colorectal cancer, endometrial cancer or pancreatic cancer^17–19^. And a study reported the NPS was of great prognostic significance in stage I–II NSCLC patients who underwent completely VATS lobectomy^34^. However, the association between the NPS and advanced NSCLC patients remains unclear. Therefore, we focused on the prognostic value of NPS in NSCLC patients with different TNM stages. To our knowledge, this is the first study to completely investigate the association between pretreatment NPS and prognosis in NSCLC patients.

Our multivariable analysis suggested that NPS was an independent prognostic factor for OS and PFS in NSCLC patients. And compared with a single indicator (including albumin, total cholesterol, NLR and LMR) and previous scoring system (including PNI, CONUT and SIS), the NPS turned to be more reliable for the prognosis of NSCLC. Considering that the TNM staging system is the main reference of the prognostic assessment in NSCLC patients, and that patients at the same TNM stage may still have different clinical outcomes, other factors are needed to further identify the prognosis for patients in the same TNM stage. We further performed a subgroup analysis of NPS and NSCLC survival according to different TNM stages and showed that NPS was an independent prognostic factor for OS and PFS in early (I–IIIA) stage patients. This was consistent with findings from the study by Li et al.^34^. Besides, our findings suggested that NPS could be also applied to predict the prognosis of advanced (IIIB–IV) stage NSCLC.

In addition, our results demonstrated that the effect of NPS on prognosis differed according to smoking status, particularly among former/current smokers. Several studies have indicated that tobacco smoke activates epithelial cells and macrophages and induces pulmonary inflammation and secretion of inflammatory cytokine and chemokine, which promotes lung cancer cell proliferation and migration^35,36^. Besides, a recent research suggested that previous or current waterpipe smoking was associated with changes in lipid metabolism, including elevated LDL-C and total cholesterol levels^37^, indicating that smoking may disturb the balance of nutrition and immune response. Further studies are needed to evaluate the association between smoking and inflammatory and nutritional status and how their potential interaction may affect the survival of patients with smoking-related cancers.

The mechanism by which high NPS contributes to a poor prognosis in patients with NSCLC is unclear. Neutrophils are recruited to tumor sites via chemokines and cytokines, and they subsequently release factors that remodel the extracellular matrix in the tumor microenvironment or act directly on tumor cells to enhance tumor proliferation and invasion^38^. Monocytes can differentiate into tumor-associated macrophages that increase angiogenesis, enhance tumor cell mobility and invasiveness, and therefore accelerate tumor cell intravasation and systemic tumor cell dissemination^39^. Unlike neutrophils and monocytes, lymphocytes have a central role in anti-tumor immunity by inducing cytotoxic cell death and inhibiting tumor cell proliferation and migration^40^. And lymphopenia may contribute to aggressive tumor biology, cancer progression, and a poor prognosis^41^. Albumin, a serum protein with greatest abundance, is responsible for maintaining colloid osmotic pressure and may influence microvascular integrity and aspects of the inflammatory pathway. Thus, the reduction of serum albumin is a sign of both inflammation and malnutrition^42^. Cholesterol is an important lipid for maintaining cellular homeostasis. Low cholesterol may impair function of the immune system, increase susceptibility to oxidative stress, induce production of cancer-related inflammation factors^43^. A recent study also suggests that a high serum cholesterol level reinforces the anti-tumor ability of NK cells and thus protects against cancer progression^44^. Thus, an elevated NPS, which is characterized by low levels of albumin, total cholesterol and NLR while a high level of LMR, is likely a reflector of more serious inflammation and a poorer nutrition status in patients.

This study has several strengths, including the prospective design to minimize the potential for reverse causality. Furthermore, to the best of our knowledge, this is the first study to explore of the relationship between pretreatment NPS and NSCLC survival. Nevertheless, several limitations should be acknowledged in current study. First, the sample size was relatively small. There were only 46 patients in NPS Group 0, resulting in limited or no outcome events in several subgroups. Thus, the findings of the present study may need to be generalized with caution, and further studies with larger sample sizes are needed to precisely validate these results. Second, there was a lack of complete data collections regarding functional status (e.g., Karnofsky and ECOG performance status scoring) and gene mutation (e.g., EGFR, KRAS and ALK) that may influence tumor progression. Therefore, the influence of above potential confounders or effect modifier could not be excluded and studies integrating these indicators could contribute to further understanding of the prognostic factors for NSCLC patients.

## Conclusion

Our findings showed that a higher NPS was substantially associated with poorer survival among NSCLC patients. Further studies are needed to validate our findings and to further explore the potential role of smoking in inflammation- and nutrition-mediated prognosis among NSCLC patients.

## Supplementary Information

Supplementary Figure S1.

## Data Availability

The data that support the findings of this study are available on request from the corresponding authors. The data are not publicly available due to privacy or ethical restrictions.
