# Supplementary material for: The prognostic value of the Naples prognostic score for patients with non-small-cell lung cancer
Source: Sci Rep. 2022 Apr 6;12:5782. doi: 10.1038/s41598-022-09888-1 (PMC8986824; doi:10.1038/s41598-022-09888-1)
Supplement: Supplementary file 1 — Supplementary Figure S1. [file 41598_2022_9888_MOESM1_ESM.docx]

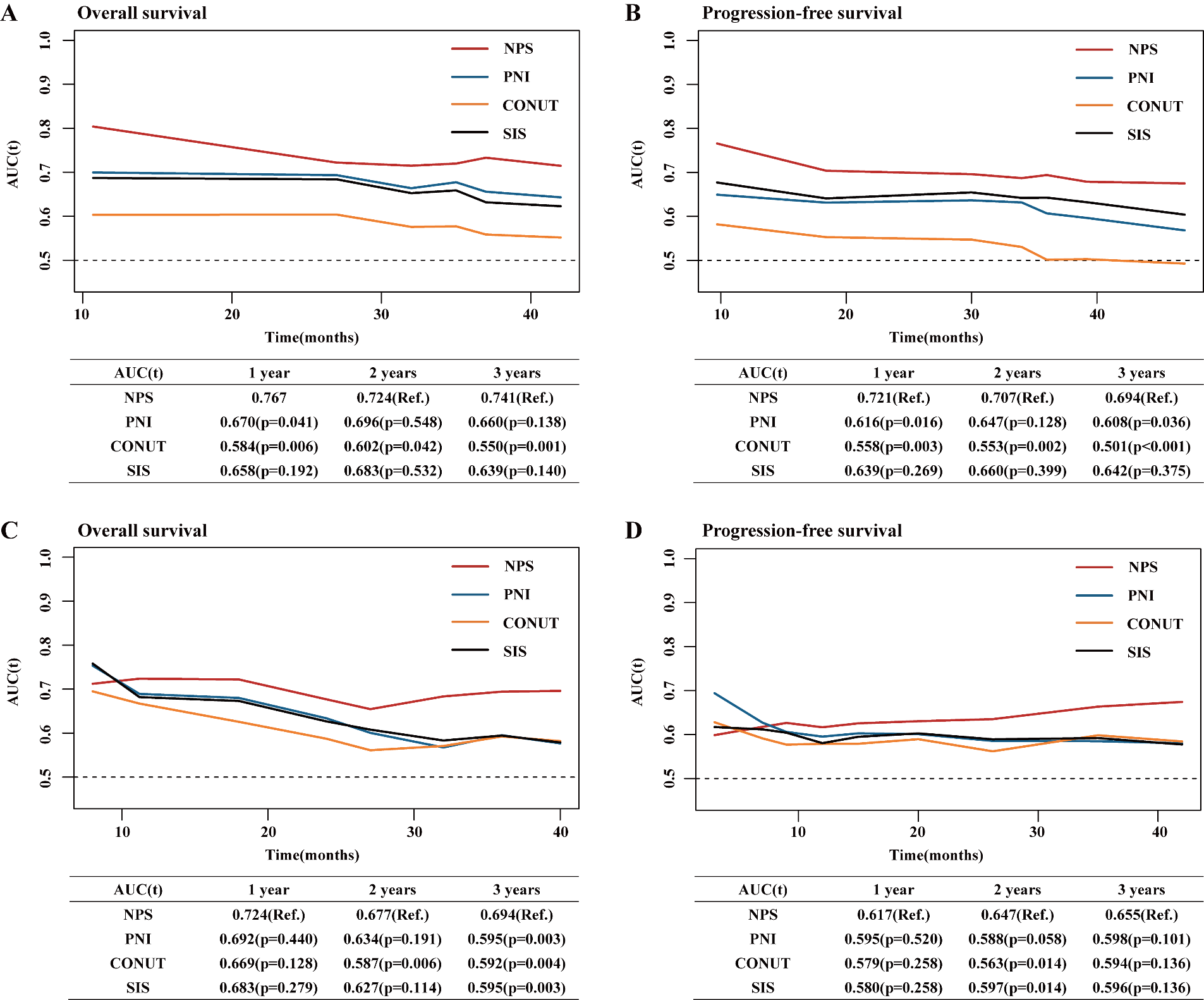


**Supplementary Fig. S1** Time-dependent receiver operating characteristic curves of NPS, PNI, CONUT and SIS for prediction of survival. The time dependence of each area under the curve (AUC) for survival is shown at 1, 2 and 3 years. (**A**) Overall survival (OS) in stage I-IIIA. (**B**) Progression-free survival (PFS) in stage I-IIIA. (**C**) OS in stage IIIB-IV. (**D**) PFS in stage IIIB-IV. CONUT, controlling nutritional status; NPS, Naples prognostic score; PNI, prognostic nutritional index; Ref., reference, SIS, system inflammation score.
